# Acknowledgments

**DOI:** 10.4269/ajtmh.1114siii

**Published:** 2024-10-01

We are extremely grateful for all those who supported the initiation of this multi-country trial and the collective presentation of the findings in this American Journal of Tropical Medicine and Hygiene (AJTMH) Supplement. The initial concept for these studies was supported by Emily Wainwright (USAID), Jonathan King (WHO), the late Vasanthapuram Kumaraswami (ICMR, Chennai, India), and the late Mwele Malecela (WHO and NIMR, Tanzania). We are also grateful for the assistance of the DMSBs for the two major research groups involved in this five-country trial.

The study team would also like to acknowledge the editorial staff at AJTMH, especially Editor-in-Chief Phil Rosenthal, Managing Editor Alison Jaeb, and Editorial Production Manager Annie Calacci for their constant attentive support throughout the preparation of this Supplement. Additionally, we would like to thank the guest editor, Jim Kazura, for his encouragement and oversight of the peer-review process.

Financial support: This supplement was supported by United States Agency for International Development (USAID) through its Neglected Tropical Diseases Program through their support of the Coalition for Operational Research on Neglected Tropical Diseases (COR-NTD) grant (AID-OAA-G-14-00008). The trials and their presentation were a collaborative effort between COR-NTD (Task Force for Global Health, Atlanta, USA) and IMMIP (Institute of Medical Microbiology, Immunology and Parasitology, The German Center for Infection Research, Bonn-Cologne site, Germany) with financial support from The German Federal Ministry of Education and Research (BMBF).

Disclosure: The views expressed herein are those of the author(s) and do not necessarily reflect the views of their institutions.

